# Measuring Communication in Microbial Biofilms in Response to Antibiotics, Phytochemicals and Stressors

**DOI:** 10.3390/antiox15030361

**Published:** 2026-03-12

**Authors:** Jean-Marc Zingg, Pratibha Joshi, Michael Moraskie, Mengrui Li, Sherwin Reyes, Md Harun Or Roshid, Sapna Deo, Sylvia Daunert

**Affiliations:** 1 Department of Biochemistry and Molecular Biology, Miller School of Medicine, University of Miami, Miami, FL 33136-6129, USA; pjoshi@med.miami.edu (P.J.); moraskiemichael@gmail.com (M.M.); mxl1317@miami.edu (M.L.); docsnreyesmt@gmail.com (S.R.); mxr1809@miami.edu (M.H.O.R.); sdeo@med.miami.edu (S.D.); 2 Dr. John T. Macdonald Foundation Biomedical Nanotechnology Institute, University of Miami, Miami, FL 33146-2101, USA; 3 University of Miami Clinical and Translational Science Institute, University of Miami, Miami, FL 33136-6129, USA

**Keywords:** biofilm, microbial whole cell biosensor, quorum sensing, bacterial communication, stress, antibiotics, free radicals, Fenton reaction

## Abstract

A high-throughput assay system is developed for measuring communication in microbial biofilms in a 96-well microtiter plate format. In this assay, bioluminescent microbial whole cell biosensor systems (MWCBs) for quorum-sensing molecules (QSMs) are embedded into biofilms, and their response to chemical cues relevant for bacterial communication is assessed. For measuring the response to stress, a sigma factor 54 (σ^54^, RpoN)-dependent MWCB was developed. Biofilms generated in this platform were exposed to gradients of communication signals (QSMs such as N-acetyl-homoserine lactones (AHLs), 3,5- dimethylpyrazin-2-ol (DPO), or phytochemicals that can act as natural quorum-sensing inhibitors (QSIs) such as curcumin or 3,3′-diindolylmethane (DIM)), and the response pattern was monitored. Further, the regulatory role of stressors such as oxidants (H_2_O_2_) or antibiotics (ciprofloxacin, trimethoprim/sulfamethoxazole) on the communication response is assessed. QSMs induced the MWCBs at 1 h and 4 h in biofilms, but high concentrations inhibited them at 24 h. Curcumin and DIM at higher concentrations lead to inhibition of quorum sensing in biofilms after 4 h and 24 h, but this is not followed by biofilm disintegration. H_2_O_2_ above 0.002% efficiently inhibited the MWCB activities and led to biofilm disintegration. At lower concentrations of H_2_O_2_, we observed induction of MWCBs. The antibiotics inhibited the MWCB activity at concentrations above their minimal inhibitory concentration (MIC), but this did not necessarily lead to disintegration of the biofilm. Like low concentrations of H_2_O_2_, the antibiotics activated the MWCBs at concentrations close to their MIC, possibly as a result of H_2_O_2_ generated during their bactericidal action. Interestingly, the induction of communication in response to antibiotics can be quenched by iron chelators, suggesting involvement of H_2_O_2_ and free radicals generated by the Fenton reaction. We hypothesize that the observed response to these stressors reflects increased communication in the biofilm, possibly enhancing tolerance and increasing survival.

## 1. Introduction

In their natural environment, bacteria can grow in biofilms that are often the cause of increased antibiotic tolerance, resistance, and pathogenicity in wounds, infectious diseases and implantable devices [1,2]. Natural biofilms are complex systems consisting of mixed-species communities with high cell density ranging from 10^8^ to 10^11^ cells per gram wet weight [3]. Further, they consist of extracellular polymeric substances (EPSs) and extracellular DNA (eDNA) that support bacterial adherence and provide biofilm structure. Emergent biofilm properties that are developed by the community when grown in biofilms involve EPS affecting sorption, enzyme retention, cooperation, communication, competition, tolerance and resistance. Within the biofilm, the concentrations of nutrients, metabolites, polysaccharides, proteins/enzymes, lipids, extracellular DNA (eDNA), and environmental molecules (organic/inorganic) are often different when compared to planktonic culture. The generally negative charge of the EPS can entrap many molecules, such as positively charged antibiotics, and make the bacteria more tolerant to antibiotics, pharmaceuticals, chemicals (e.g., toluene and xylene) and desiccation. Depending on the bacteria and antibiotics, biofilms can tolerate up to 100–1000 times higher concentrations of antibiotics and disinfectants when compared to planktonic cells [4].

The communication mechanisms between bacteria are also different in biofilms when compared to planktonic cultures. Bacterial quorum-sensing molecules (QSMs) are the molecules bacteria use for population density-dependent communication, a process referred to as quorum sensing (QS) [5,6,7,8]. In biofilms, the concentrations of QSMs, such as acyl-homoserine lactones (AHLs), have been estimated to be 1000-fold higher when compared to planktonic cells [3]. QSMs are well known to facilitate the transition from planktonic culture into biofilms and contribute to increased pathogenic behavior, adherence, secretion, immune subversion, biofilm formation, toxin production, and resistance to both antimicrobials and biological stressors [9,10,11]. Natural or synthetic quorum-sensing inhibitors (QSI) that interfere with bacterial communication have been proposed as novel strategies to combat biofilms and antibiotic resistance [12,13,14]. However, it has been challenging to develop assays that can measure changes in signaling and communication within the biofilm and to assign a physiological function to these changes [3,14,15,16].

A number of assays have been developed for assessing microbial biofilm formation and eradication [17,18,19,20,21,22,23,24]. The standard staining test uses crystal violet or similar dyes, such as safranin, that quantify the dye bound to bacteria and EPS on polystyrene and other hydrophobic substrates [25,26,27]. Closed-system methods include the microtiter plate method, the tube method, or the Congo red agar method. Dynamic open systems methods with continuous culture include the drip flow reactor, the rotary biofilm reactor, the flow chamber method, and microfluidic devices. Imaging methods include light microscopy (LM), atomic force microscopy (AFM), confocal laser scanning microscopy (CLSM), and scanning electron microscopy (SEM). Most of these methods do not allow simultaneous testing for multiple conditions, with the exception of microtiter plate-based methodologies. A variant of the microtiter plate method is the Calgary method, in which the bacteria adhere to polystyrene PEGs hanging in the microtiter plate, and the bacteria adhere to the PEGs from below [4,28,29,30]. This arrangement prevents clustering and clumping of sedimented bacteria that collapse at the bottom of the well, which can lead to artifacts by the microtiter plate method. The biofilm is stained with Gram crystal violet or safranin, followed by extraction, and then the absorbance was measured. These cationic dyes not only stain viable bacteria, but also stain dead cells and some of the negatively charged components of the EPS via ionic interactions. Based on the Calgary method, several pharmacodynamic parameters of antimicrobial activity in planktonic and biofilm-growing bacteria have been defined to compare the activity of antibiotics (minimal inhibitory concentration (MIC), minimum biofilm inhibitory concentration (MBIC), minimum bactericidal concentration (MBC), minimum biofilm eradication concentration (MBEC), biofilm prevention concentration (BPC)) [4], and a number of methods have been developed to assess the viable cell counts in biofilms [30].

Here, we have developed a novel assay system using bioluminescent microbial whole cell biosensor systems (MWCBs) that allows us to monitor the response of bacteria in biofilms to QSMs and chemical cues in a high-throughput 96-well microtiter plate format. We have modified the existing platform for measuring biofilm formation, the MBEC assay (Innovotech), which is used to determine the minimum biofilm eradication concentration (MBEC) and is based on the Calgary biofilm method [31]. In addition to endpoint staining with Gram crystal violet, we detect microbial communication by measuring emission of bioluminescence by MWCBs embedded into the biofilm in response to cues such as QSMs, QSIs, antibiotics, oxidants, and other stressors in a concentration- and time-dependent manner. Sender and receiver bacteria are used to monitor communication in biofilms between two types of bacteria of the same or different species. In addition to being able to detect bacterial communication in biofilms in a rapid and multiplex format, the assay has the potential to be fully automatable.

## 2. Materials and Methods

### 2.1. Materials

N-acetyl-homoserine lactones (AHLs) and 3,5- dimethylpyrazin-2-ol (DPO) (Sigma-Aldrich, St. Louis, MO, USA) were dissolved as 50 mM stocks in acetonitrile. Curcumin (95% total curcuminoid content, Alfa Aesar, Waltham, MA, USA) and 3′-3′-diindolylmethane (DIM) (Sigma-Aldrich) were dissolved in ethanol as a 50 mM stock. H_2_O_2_ (Sigma-Aldrich) was freshly diluted before each use.

### 2.2. Bacteria

*E. coli* NEB^®^5α (*fhuA2::IS2*, *Δ*(*mmuP-mhpΔ*)*169*, *ΔphoA8*, *glnX44*, *Δ80d[ΔlacZ58(M15)]*, *rfbD1*, *gyrA96*, *luxS11*, *recA1*, *endA1*, *rph^WT^*, *thiE1*, *hsdR17*) was purchased from New England Biolabs (NEB) [32]. For biofilm assays, *E. coli* JM109 (*endA1*, *recA1*, *gyrA96*, *thi*, *hsdR17 (rk^–^*, *mk^+^*), *relA1*, *supE44*, *Δ(lac-proAB*), [*F’ traD36*, *proAB*, *laqI^q^ZΔM15*] was purchased from Promega (Madison, WI, USA). For assays with DPO, a *tdh*-knockout *E. coli* strain (SP942, JW3591-4) that does not produce DPO (derived from *E. coli* K-12 BW25113, Keio collection, Yale *E. coli* genetic stock center, https://cgsc.biology.yale.edu/, accessed on 19 August 2019) was used. Sigma factor 54-deficient *E. coli* (ΔrpoN730::kan(Δσ^54^) was from the Yale *E. coli* genetic stock center. Bacteria were grown in LB media, and assays were done in yeast extract media (5 g yeast extract and 10 g NaCl per liter) that gave consistent results with the biosensors.

### 2.3. Transformation of Bacteria by Electroporation

Electrocompetent bacteria were generated by growing them to an OD_600_~0.6, centrifugation and the pellet was taken up in 20 mL ice cold 10% glycerol. Centrifugation and resuspension was repeated 4 times. Finally, the pellet is taken up in 250 μL ice cold 10% glycerol and either used directly or aliquoted and stored at −80 °C. For electroporation, 1 μL plasmid (~100 ng) is added to 50 μL electrocompetent bacteria and transferred to an electro cuvette (Biorad, Hercules, CA, USA, 0.2 cm gap). Electroporation is performed at 2500 V for about 5.8 ms using an Eppendorf electroporator (Eppendorf SE, Hamburg, Germany). The bacteria are taken up with 1 mL LB media and grown for 1 h at 37 °C for recovery, and then spread on agar plates.

### 2.4. Construction of Novel MWCBs Plasmids

A number of plasmids were generated for this study; more details are given in the Supplementary information.

*Plasmid construction of pPsmut for measuring the σ^54^-dependent stress response.* We have engineered and characterized a novel MWCB, pPsmut, for the detection of the σ^54^-dependent stress response as a result of exposure to oxidants, antibiotics, xylenes and related organic compounds. This MWCB takes advantage of a mutant transcriptional activator of *XylR* (GenBank: M10143.1), *XylR28*, and a truncated version of the corresponding operator/promoter region taken from the *Xyl* operon from *Pseudomonas putida* (Figure S1) [33,34,35,36]. The mutated XylR28 protein is under the control of the Pr1/2 promoters and the LuxCDABE cassette from *Photorhabdus luminescence* is under the control of the XylR-inducible Ps1 promoter. The construct (pPsmut) contains a truncated version of the Ps promoter containing only the σ^54^-controlled Ps1 promoter but not the σ^70^-controlled Ps2 promoter that is regulated by XylR and controls the expression of the luxCDABE reporter cassette.

*Plasmid construction of pPsmutdeltaE.* During sequencing of the XylR gene from plasmid XylR28, it was noted that it contained an additional EcoRI site at the 5′-end generated during construction that extended the amino acid sequence by two amino acids (GluPhe) [37]. Analysis of the protein structure of XylR28 using Alphafold (version 2.0) revealed that these two additional amino acids may not change the overall structure of XylR28, but it appeared possible that they may affect the A domain that acts as an intramolecular repressor and shifts its orientation during activation by ligand binding [38]. Therefore, these two amino acids were deleted. Like pPsmut, pPsmutdeltaE allows for detection of the σ^54^-dependent stress response, but generally gives a higher output in bioluminescence.

*Plasmid construction of pVIBdeltaI.* The luciferase cassette of *Aliivibrio fischeri* (plasmid pJE202, [39]) was modified by deleting the LuxI gene that produces C6-AHL. The construct contains the *Aliivibrio fischeri* luciferase operon (LuxCDABEG) without LuxI and is inducible by AHLs via LuxR (Figure S2A).

*Plasmid construction of pSmutVIBI.* The LuxI gene of *Aliivibrio fischeri* (plasmid pJE202, [39]) was inserted into pSmut. The construct contains the *Aliivibrio fischeri* LuxI gene expressed by the Ps1 promoter of plasmid pSmut (Figure S3).

*Plasmid construction of pETVIBI.* The LuxI gene of *Aliivibrio fischeri* (plasmid pJE202, [39]) was inserted into pET29b(+). The construct contains the *Aliivibrio fischeri* LuxI gene controlled by the lac-operator repressed T7 promoter and can be induced by isopropyl-beta-D-thiogalactopyranoside (IPTG) (Figure S3).

*Plasmid construction of pSigma54.* The *ntrA* gene (σ^54^ or *rpoN*) (GenBank: M24916.1) was amplified from *Pseudomonas putida* KT2440 (ATCC^®^ 47054™) genomic DNA and inserted into pBR322 (NEB). We chose pBR322 as the plasmid with a p15A origin of replication to be compatible when transformed into the same host, with the pGEN-LuxCDABE-derived plasmids that are based on ColE1 origin of replication. The construct contains the *Pseudonomas putida ntrA* gene (σ^54^) expressed under control of its own promoter.

### 2.5. Assay for Measuring Bacterial Communication in Biofilms

We have adapted the MBEC assay (Innovotech, Edmonton, AB, Canada), which is used to determine the minimum biofilm eradication concentration and is based on the Calgary biofilm method [31]. *E. coli* JM109 was transformed with the MWCB plasmids, and a single colony was picked and grown at 37 °C overnight. For the assays, the overnight culture (OD_600_ ~1.2–1.4) was diluted 1/100 with media, and 150 μL was added to the PEG plate and grown at 37 °C for 20–24 h, with shaking at 110 rpm in a humidified atmosphere. The PEG plate was then inserted into a black microtiter plate with a transparent bottom, inserted into the microtiter plate reader with the PEG pointing upwards, and bioluminescence was measured using top reading (0 h untreated control reading) (Clariostar, BMG Labtech, Ortenberg, Germany). Measurement can also be made without a black plate, but it can increase the spill-over of signals from neighboring PEGs and affect the results, especially when the signal strength varies a lot. Then the PEG plate was inserted into a new microtiter plate containing the various experimental treatments specified in the figure legends in 150 μL fresh media, and the plate was incubated in a humidified atmosphere at 37 °C for 1 h, 4 h, and 24 h, with shaking (110 rpm). After that, the bioluminescence was detected as described above, and the relative light units (RLU) representing the activity of the MWCBs in the biofilm were plotted.

### 2.6. Gram-Crystal Violet Staining

For staining of the biofilms on PEGs, 150 μL of Gram crystal violet solution (GramCV, Sigma, St. Louis, MO, USA) was added to a microtiter plate, the PEG plate was inserted, and it was shaken at room temperature for 20 min. Then, the plate was washed 3× in 200 μL PBS, photographed, and eluted with 200 μL 95% ethanol. The absorbance of the eluted dye was measured at 595 nm (Clariostar, BMG Labtech). Generally, biofilm formation was quite equal over the entire plate unless bactericidal treatments were used.

### 2.7. Paper Disk and Antibiotic Strips Assays

In total, 20 µL of H_2_O_2_ freshly diluted at various concentrations was added to Grade AA Whatman disks and positioned onto a bacterial lawn for detecting the response of the MWCBs. These disks are routinely used to assess the antibiotic resistance of bacteria for checking the sensitivity to antibiotics and chemotherapeutic agents in vitro by means of the inhibition zone determination method (the Kirby–Bauer method) [40,41,42]. Similar experiments were done with test strips containing gradients of antibiotics (ETEST, Biomerieux, Hazelwood, MO, USA).

### 2.8. Statistics

All experiments were performed in quadruplicate and performed at least twice to ensure repeatability. All values were calculated as the mean ± standard error of the mean (SEM) as explained in the figure legends.

## 3. Results

### 3.1. Development of a High-Throughput Assay for Monitoring Bacterial Communication in Biofilms

**Figure 1:**
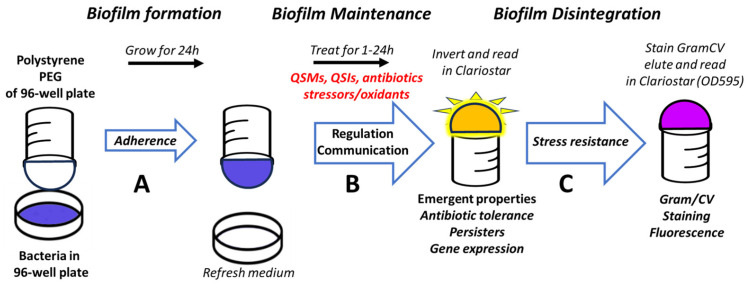
Assay principle of the HTS assay for measuring regulatory effects of chemical cues (e.g., QSMs, QSIs, antibiotics, phytochemicals, oxidants) on bacterial communication in biofilms. Depending on the timepoint at which these chemicals are added during biofilm formation, the assay measures their impact on adherence (A), maintenance (B), and disintegration (C) of biofilms. In this study, we treated the biofilms at an early stage and recorded the bioluminescent response after 1 h, 4 h and 24 h, thus reflecting communication occurring during biofilm maintenance. QSMs, quorum sensing molecules; QSIs, quorum sensing inhibitors.

To measure communication in microbial biofilms in response to antibiotics, phytochemicals, and stressors, we adapted an existing platform for measuring biofilm formation (MBEC assay with polystyrene PEGs, Innovotech) that is based on the Calgary biofilm device [31], for use with MWCBs that emit bioluminescence in response to chemical cues and communication molecules [35,36,43,44]. In a first step, the assay conditions for biofilm formation and measurement of bioluminescence with the MWCBs in biofilms were tested and optimized. Growing the biosensor plasmids with the *E. coli* strain NEB5α did not efficiently produce biofilms on the polystyrene PEGs, so we switched to strain JM109, which is often used in *E. coli* biofilm studies [45,46]. We routinely grew the bacteria overnight from a single colony (OD_600_ ~1.2–1.4) and distributed 150 μL of a 1/100 diluted culture into the 96-well plate, and then inserted the top plate containing the PEGs. The bacteria were then grown at 37 °C for 24 h with shaking (110 rpm) in a humidified atmosphere that reduces evaporation. After 24 h, we inserted the top plate with the PEGs into a black 96-well microtiter plate with a transparent bottom, inverted the plate, and measured the bioluminescence using a microtiter plate reader and top reading to give the 0 h untreated control values. At this time point, biofilms have been established, and the subsequent measurements reflect communication relevant for biofilm maintenance. The various treatments were then added to a fresh bottom plate containing fresh media, the PEG plate reinserted, and the bioluminescence of the PEG plate was measured as described above after 1 h, 4 h and 24 h. As a control for biofilm formation, after 24 h, the biofilm on the PEGs was stained with Gram crystal violet (GramCV), and after washing the biofilm with PBS and elution with 95% ethanol, the formation of biofilms was assessed by measuring OD_595_ using a microtiter plate reader. The 96-well microtiter plate high-throughput format allows for performing the assay in multiple replicates with several treatments in the same plate (e.g., quadruplicates of each treatment and condition), relevant for analysis of experiments with inherent biological variability such as biofilm formation. Using this assay, bacterial communication during adherence, maintenance, and disintegration of biofilms can be measured (Figure 1).

### 3.2. Adaptation of MWCBs Plasmids to Be Embedded into the Biofilms

**Figure 2:**
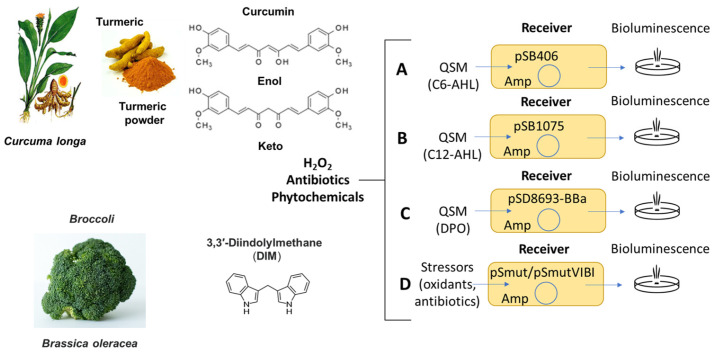
Structure of phytochemicals and plasmids constructed and adopted in this study. (**A**) pSB406, sensor for C6-AHL [47]; (**B**) pSB1075, sensor for C12-AHL [47]; (**C**) pSD8693-BBa, sensor for DPO [43]; (**D**) σ^54^-regulated pSmut and pSmutVIBI, sensors of aromatics and stressors (this study). Amp: ampicillin.

To measure bacterial communication in biofilms in response to antibiotics, phytochemicals and stressors with our assay system, we adapted previously described MWCBs to monitor QSMs (Figure 2). When incorporated as a sentinel into a microbial community such as a biofilm, these MWCB plasmids monitor activation by specific analytes and stressors and give information on how the bacterial community develops, responses and adapts. We analyzed the response of the MWCBs to externally added communication signals, namely QSMs and QSIs, once the biofilm has been formed on the PEGs, thus reflecting communication relevant for biofilm maintenance (Figure 1).

MWCBs for N-acetyl-homoserine lactones (AHLs). AHLs are well-studied QSMs used for interspecies communication by both Gram-positive and Gram-negative bacteria and regulate diverse behaviors, such as the formation of biofilms and the production of pathogenic virulence factors. To monitor bacterial communication by AHLs in biofilms, we have tested two previously described plasmids, namely plasmid pSB406 for short-chain C6-AHLs (scAHLs) (Figure 2A) and plasmid pSB1075 for long-chain C12-AHLs (lcAHLs) (Figure 2B) [47,48,49,50]. These plasmids were transformed into E. coli JM109 that do not produce AHLs but can sense AHLs through its SdiA gene [51,52]. When AHLs are added externally to these MWCBs, the AHLs bind to the regulatory protein (LasR) and activate the expression of the luciferase reporter, and the bioluminescent signal emitted is proportional to the levels of AHLs added and thus reports on the communication occurring within the bacterial community. In previous studies, we have used these MWCBs to measure QSMs in inflammatory bowel disease (IBD), spinal cord injury and depression [5,48,50,53,54]. These sensing systems allowed detection of QSMs with a limit of detection (LOD) of 1 × 10^−9^ M and a dynamic range of 1 × 10^−9^–1 × 10^−6^ M.

### 3.3. Response to Treatments with QSMs C6-AHL and C12-AHL

To demonstrate that the developed assay system can monitor communication in biofilms, we have measured the response of MWCBs in biofilms to select quorum-sensing molecules (QSMs), namely, C6-AHL and C12-AHL, in a time-dependent manner.

**Figure 3:**
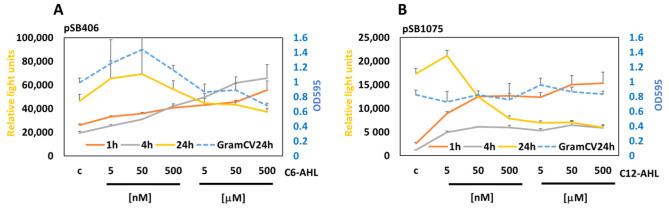
Measuring the response to scAHL and lcAHL in biofilms. MWCBs plasmids for shAHL (pSB406) (**A**) or lcAHL (pSB1075) (**B**) were embedded into biofilms and treated with increasing concentrations of C6-AHL or C12-AHL, and the bioluminescence was measured at 1 h, 4 h, and 24 h. GramCV staining (OD_595_) was used to measure the biofilm integrity after treatment for 24 h (blue stippled lines).

The treatment of the MWCB (pSB406) with C6-AHLs induced bioluminescence in the biofilm at 1 h, 4 h, and 24 h, but reduced it with high concentrations (>50 nM), leading to “*Quorum Silencing*” at 24 h in parallel with inhibition of the biofilm as detected by Gram crystal violet staining (Figure 3A).

The treatment of the MWCB (pSB1075) with C12-AHLs induced bioluminescence in the biofilm at 1 h, 4 h, and 24 h, but reduced it at high concentrations (>5 nM) at 24 h. Interestingly, the biofilm remained intact over all concentrations tested, suggesting inhibition of QS within the biofilm in a concentration-dependent manner, with high concentrations leading to “*Quorum Silencing*” (Figure 3B).

To evaluate whether the regulatory effects of QSMs other than AHLs can be assessed by our assay, we tested 3,5- dimethylpyrazin-2-ol (DPO) with our recently developed MWCB for DPO detection using our biofilm assay (Figure 2C) [43]. We were able to measure a response to DPO in a concentration-dependent manner, whereas no response was observed with control AI3. AI3 is a structural isomer of DPO and is expected not to activate the sensor for DPO (Figure S5).

### 3.4. Response to Treatments with Phytochemicals That Can Act as QSIs

Using the developed assay system, we have measured the effects of select natural quorum-sensing inhibitors (QSIs) of AHLs, namely, the phytochemicals curcumin and 3,3′-diindolylmethane (DIM). Curcumin, also known as diferuloylmethane, is present in dried turmeric powder from the rhizome of *Curcuma longa* L. as a major bioactive component, and it can inhibit growth and biofilm in a number of bacteria [55,56]. DIM is a bioactive phytochemical derived from the digestion of indole-3-carbinol found in cruciferous vegetables (e.g., broccoli, brussels sprouts, cabbage, and kale), and is known to inhibit biofilm formation, e.g., by cariogenic *Streptococcus mutans* and *Pseudomonas aeruginosa* [57,58,59].

**Figure 4:**
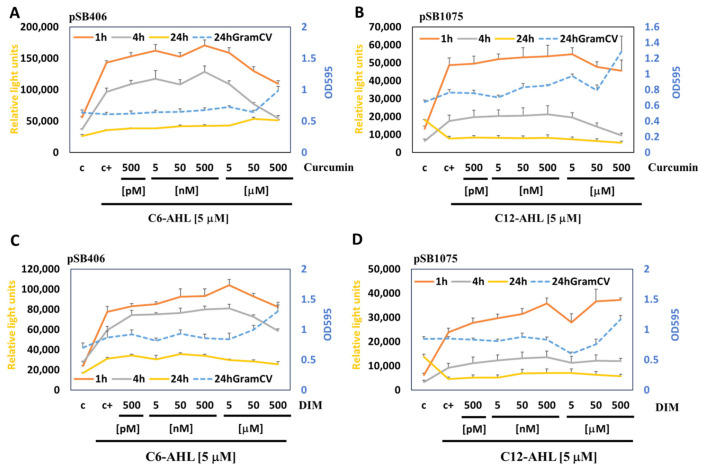
Measuring the response to the quorum-sensing inhibitor curcumin and DIM in biofilms. MWCBs plasmids for shAHL (pSB406) (**A**,**C**) or lcAHL (pSB1075) (**B**,**D**) were embedded into biofilms and treated with 5 μM C6-AHL or C12-AHL, respectively, and increasing concentrations of curcumin (**A**,**B**), and DIM (**C**,**D**), the bioluminescence measured at 1 h, 4 h, and 24 h. GramCV staining (OD_595_) was used to measure the biofilm integrity after treatment for 24 h (blue stippled lines). Note that the response to AHLs (5 μM) is silenced after treatment for 24 h.

The treatment of the MWCB (pSB406) induced with 5 μM C6-AHL and with low concentrations of curcumin increased bioluminescence but reduced it at higher concentrations (>500 nM) at 1 h and 4 h. The higher staining by Gram crystal violet when treated with 500 μM curcumin is likely the result of interference with absorbance at OD_495_ nm by curcumin extracted from the biofilm (Figure 4A).

The treatment of the MWCB (pSB1075) induced with 5 μM C12-AHL and increasing concentrations of curcumin reduced bioluminescence only at high concentrations (>5 μM) at 1 h and 4 h. No response could be measured at 24 h as a result of inactivation of the sensor by “*Quorum Silencing*”. The higher staining when treated with 500 μM curcumin is likely the result of interference with absorbance at OD_495_ nm by curcumin extracted from the biofilm (Figure 4B).

The treatment of the MWCB (pSB406) induced with 5 μM C6-AHL and increasing concentrations of DIM reduced bioluminescence only at high concentrations (>5 μM) at 1 h and 4 h. A weak increase was observed at lower concentrations at 1 h and 4 h. No response could be measured at 24 h as a result of inactivation of the sensor by “*Quorum Silencing*”. Biofilm formation measured by Gram crystal violet staining was increased above 5 μM (Figure 4C).

The treatment of MWCB (pSB1075) induced with 5 μM C12-AHL and increasing concentrations of DIM did not decrease bioluminescence, but a weak increase was observed at low concentrations at 1 h and 4 h. Biofilm formation measured by Gram crystal violet staining increased at 500 μM (Figure 4D).

Taken together, we observed that curcumin and DIM at higher concentrations lead to inhibition of quorum sensing in biofilms that is not followed by biofilm disintegration (Figure 4). Accordingly, potential binding sites for curcumin have been identified in RhlR and LasR, and inhibition of QS-regulated phenotypes was observed in *P. aeruginosa* at high concentrations (12.5 to 50 μM), but biofilms were not affected [60]. In contrast, at lower concentrations and short incubation times (1 h and 4 h), we observed increased bacterial communication, which is opposite to what was expected based on prior knowledge inferred from planktonic cultures and may depend on the specific type of bacteria that we used (*E. coli* JM109) [57,58,59].

### 3.5. Monitoring the Response of MWCBs to Stressors (H_2_O_2_) in Biofilms

We used the developed assay system to measure communication of MWCBs grown in biofilms in response to elevated oxidative stress (e.g., H_2_O_2_). To measure the bacterial stress response, a novel MWCB plasmid, pSmut, was constructed in which the luxCDABE cassette from *Photorhabdus luminescence* is controlled by the stress-responsive σ^54^-controlled Ps1 promoter of the *Pseudomonas putida* Xyl operon (Figure 2D and Figure S1). No bioluminescence is observed in the absence of σ^54^ (when transformed into *E. coli* ΔrpoN730::kan(Δσ^54^)), demonstrating that σ^54^ is absolutely necessary for expression (Figure S4). Further, overexpression of σ^54^ in this strain from pSigma54 restores expression (Figure S4). In plasmid pSmut, the Ps1 promoter is controlled by σ^54^ (*RpoN*), important for stress response and biofilm formation, leading to regulation of the luxCDABE genes [61,62], and the Pr promoter regulates the *XylR* gene able to sense organic molecules related to xylene. Therefore, pSmut is monitoring σ^54^-regulated bacterial gene expression relevant for pathogenic behavior, quorum sensing, adherence, secretion, immune subversion, biofilm formation, toxin production, and resistance to both antimicrobials and biological stressors [9,10,11,62].

**Figure 5:**
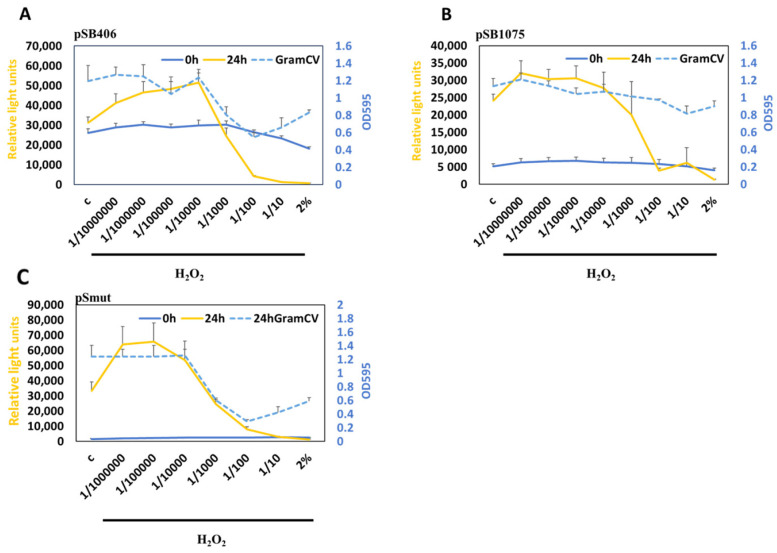
Measuring the response to the oxidant H_2_O_2_ in biofilms. MWCBs plasmids for shAHL (pSB406) (**A**), lcAHL (pSB1075) (**B**), or pSmut (**C**) were embedded into biofilms and treated with increasing concentrations of H_2_O_2_, and the bioluminescence was measured at 0 h and 24 h. GramCV staining (OD_595_) was used to measure the biofilm integrity after 24 h (blue stippled lines).

In addition to pSmut, we have analyzed the response to increasing concentrations of H_2_O_2_ with MWCBs carrying the plasmids for scAHLs (pSB406) and lcAHLs (pSB1075). We find that H_2_O_2_ above 0.002% efficiently inhibits the activity of all the biosensors tested and leads to biofilm disintegration as expected for bactericidal H_2_O_2_ (Figure 5A–C). Interestingly, at low concentrations of H_2_O_2_, we observed induction of our MWCBs that is most pronounced with pSmut.

### 3.6. Monitoring the Response of MWCBs to Stressors (Antibiotics) in Biofilms

We have used the developed assay system to measure the communication of MWCBs grown in biofilms in response to antibiotics. Antibiotics can either be bacteriostatic or bactericidal. One of the mechanisms of antibiotic action involves oxidative damage by free radicals, e.g., generated from H_2_O_2_ by Fenton chemistry [63,64]. Intracellular H_2_O_2_ increases in the presence of certain antibiotics [65], and the antimicrobial action of antibiotics may reflect the generation of ROS until bacterial cell death occurs. As a consequence, bacteria have developed systems to defend themselves against oxidants, such as H_2_O_2_ [66,67,68]. QS plays a role in developing bacterial resistance against antibiotics and H_2_O_2_ [69] that possibly can be monitored in the biofilm with our MWCBs.

**Figure 6:**
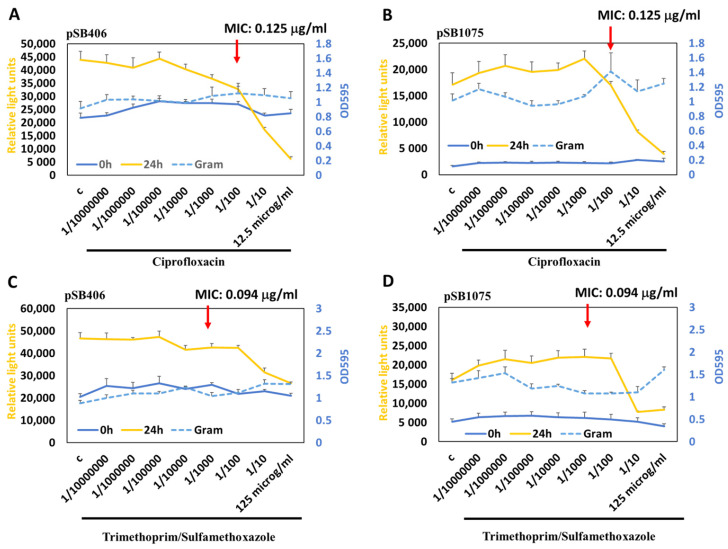
Measuring the response to the antibiotic ciprofloxacin and trimethoprim/sulfamethoxazole in biofilms. MWCBs plasmids for shAHL (pSB406) (**A**,**C**) or lcAHL (pSB1075) (**B**,**D**) were embedded into biofilms and treated with increasing concentrations of ciprofloxacin (**A**,**B**) or trimethoprim/sulfamethoxazole (**C**,**D**), and bioluminescence was measured at 0 h and 24 h. GramCV staining (OD_595_) was used to measure biofilm integrity after 24 h treatment (blue stippled lines).

Interestingly, the bactericidal antibiotics ciprofloxacin and trimethoprim/sulfamethoxazole inhibit the MWCBs activity in the biofilm at concentrations above the MIC, but the integrity of the biofilm itself is much less affected when compared to H_2_O_2_ (Figure 6A–D). These results point out that these antibiotics can inhibit signaling in the biofilm, but this does not necessarily lead to the disintegration of the biofilm within the time of the experiment.

Further, our results suggest that treatment with H_2_O_2_ may be a more useful agent to destroy biofilms than the tested antibiotics. Interestingly, similar to low concentrations of H_2_O_2_, we observed slight induction of our MWCBs with ciprofloxacin and trimethoprim/sulfamethoxazole with pSB1075 and pSB406, possibly explaining AHL-like effects we previously observed with certain antibiotics [70].

### 3.7. Monitoring the Response of MWCBs to Stressors (Antibiotics) in Bacterial Lawns

**Figure 7:**
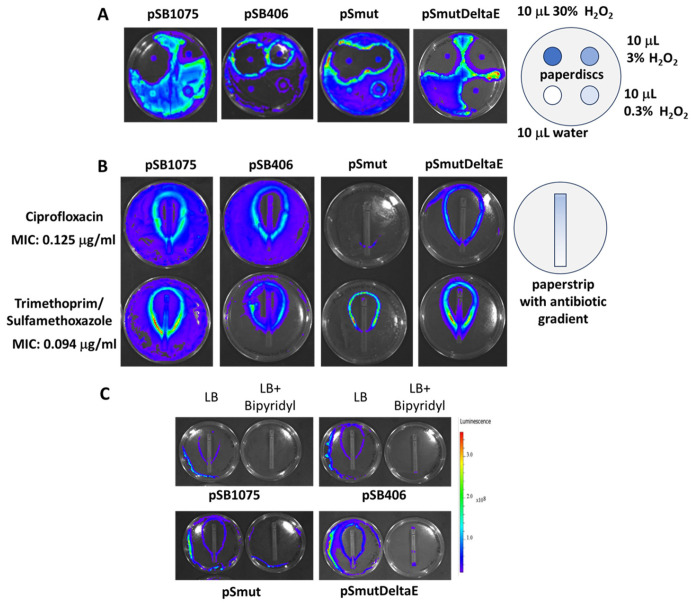
Analysis of biosensor response induced by H_2_O_2_ and antibiotics. (**A**) Paper disks containing dilutions of 3% H_2_O_2_ were placed on lawns of *E. coli* containing the MWCBs and grown overnight. (**B**) Paperstrips with antibiotic gradients were placed on lawns of *E. coli* containing the MWCBs and grown overnight. (**C**) Paperstrips with gradients of antibiotic (ciprofloxacin) were placed on lawns of *E. coli* containing the MWCBs and grown overnight on agar plates with and without the iron chelator 2,2′- bipyridyl (0.35 mM). Pictures were acquired using the IVIS system (Perkin Elmer, Waltham, MA, USA).

To better understand the signaling by H_2_O_2_ and antibiotics, we monitored bacterial lawns made up of the MWCBs and exposed to paper disks and strips containing different amounts of these stressors positioned on top (similar to the Kirby-Bauer method) [40,41]. Interestingly, we observed the activation of bioluminescent emissions in a circular manner in the defense zone at the border to the “zone of death” and paper disks with increasing concentrations of H_2_O_2_ (Figure 7A). A similar activation of the MWCBs was observed with the tested antibiotics, but the response intensity depends on the type of antibiotic (Figure 7B). When using paper strips that contain gradients of increasing concentrations of the antibiotics, the same intensity of bioluminescence was observed in the defense zone at the border to the “zone of death”, independent of the concentration of the antibiotic, suggesting that activation reflects communication of the bacteria in defense of the stressor once a threshold close to induction of cell death is reached. When the availability of Fe^2+^ in the agar plate was limited by including an iron chelator (0.35 mM 2,2′- bipyridyl [68]), a reduction in bioluminescence at the border to the “zone of death” was observed (Figure 7C), suggesting that increased production of hydroxyl radicals generated by the Fenton reaction may be involved. Likewise, the MIC for ciprofloxacin was slightly increased in the presence of the iron chelator, but since the response of bacteria to antibiotics, iron, and H_2_O_2_ can be complex and is still controversial [71], further research will be required to delineate the molecular mechanisms involved.

### 3.8. Communication in Biofilms Between Sender and Receiver MWCBs in E. coli

The above experiments showed that the assay system can monitor the response of one type of bacteria carrying MWCBs in response to QSMs, QSIs, and stressors when grown in biofilms. To investigate whether the assay system can also monitor communication between two different types of bacteria in biofilms, we have constructed Sender and Receiver plasmids, in which the Sender bacteria produce QSMs, and the Receiver bacteria respond to them [72]. To measure communication between different bacteria, the Sender plasmid can be transformed into one bacterium, and the Receiver into another one (same or different strain). The Receiver plasmid pVIBdeltaI was constructed by deleting the *luxI* gene that synthesizes C6-AHL in *Vibrio fischeri* from plasmid pJE202 (Figure S3). The Receiver plasmid pVIBdeltaI was transformed into *E. coli* strains that do not produce AHLs, and tested by adding different AHLs into the culture media and growing them for 24 h at 37 °C. Interestingly, when compared to plasmids pSB1075 and pSB406, pVIBdeltaI did not give background in the absence of AHLs. Adding increasing amounts of C6-AHL induced bioluminescence production up to 1600-fold as compared to when there are no QSMs present (Figure S2). Other AHLs showed lower induction. Of interest is the finding that the oxidized C10- and C12-AHLs (3-oxo-C10 and 3-oxo-C12 AHLs) showed stronger activation than the non-oxidized ones, suggesting that the AHL-mediated communication is modulated by stressors such as free radicals. Free radical modulated QS may occur either by directly modifying QS molecules [73], by influencing the transcription factors binding to the promoter, or by affecting the luciferase [74,75]. Oxidized AHL, such as 3-oxo-C12:2-AHL, has relevance for diseases such as ulcerative colitis by limiting cytokine-induced intestinal tight junction disruption [76].

Two Sender plasmids were constructed. The Sender plasmid (pETVIBI) produces C6-AHL and induces the Receiver (pVIBdeltaI) in response to IPTG (Figure S3). The Sender plasmid (pSmutVIBI) expresses LuxI and synthesizes C6-AHL via the Ps1 promoter (same as in pSmut) and induces the Receiver (pVIBdeltaI) when induced by stress and when grown in close contact with the Receiver (pVIBdeltaI) on agar plates containing the inducer (Figure S1).

**Figure 8:**
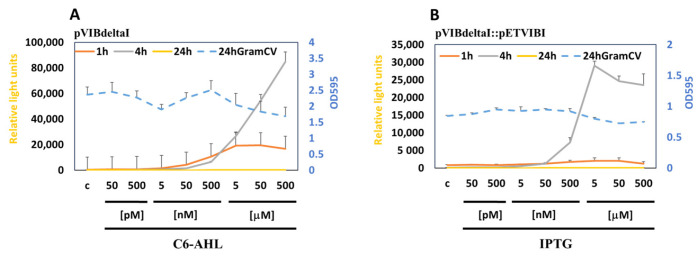
Measuring the response of Receiver bacteria to Sender bacteria in biofilms. (**A**) MWCB containing the Receiver plasmid pVIBdeltaI was integrated into biofilms and induced by increasing concentration of C6-AHL. (**B**) MWCB containing Receiver plasmid pVIBdeltaI was integrated into biofilms together with the MWCB Sender containing plasmid pETVIB and induced by increasing concentration of IPTG. Bioluminescence was measured at 1 h, 4 h and 24 h. GramCV staining (OD_595_) was used to measure biofilm integrity after 24 h treatment (blue stippled lines).

We integrated Sender and Receiver MWCBs into the biofilm (1:1 of 1/100 dilution) and monitored the response of the Receiver upon induction of the Sender. As a model, we also constructed and used the Sender pETVIBI, which produces C6-AHL upon induction by IPTG, leading to activation of the Receiver (pVIBdeltaI). As expected, the Receiver could sense the production of C6-AHL in response to IPTG, both when grown in liquid culture or lawns (Figure S4), or in biofilms (Figure 8).

### 3.9. Communication in Biofilms Between Two Different Types of Bacteria

**Figure 9:**
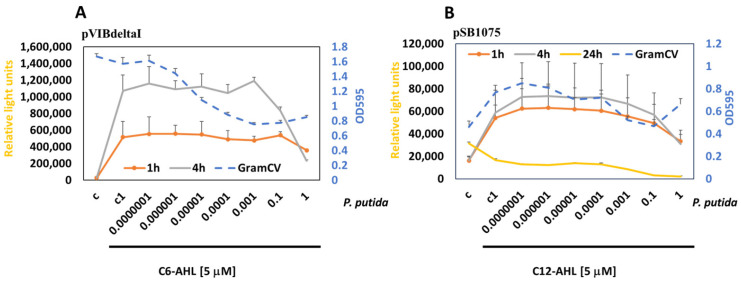
Measuring the response of Receiver bacteria in biofilms exposed to *Pseudomonas putida*. (**A**) MWCB containing the Receiver plasmid pVIBdeltaI was integrated into biofilms on the PEG and exposed to increasing numbers of *P. putida* grown planktonically in the presence of C6-AHL. (**B**) MWCB containing Receiver plasmid pSB1075 was integrated into biofilms and exposed to increasing numbers of *P. putida* grown planktonically in the presence of C12-AHL; GramCV staining (OD_595_) was used to measure biofilm integrity after 24 h treatment (blue stippled lines).

To monitor the communication that occurs when two different types of bacteria interact, we grew *Pseudomonas putida* and monitored the response in the biofilm consisting of *E. coli* carrying the MWCBs [77,78]. *P. putida* can produce AHLs of various chain lengths, but also has the ability to inhibit communication by quorum quenching [77,79]. Serially diluted samples from *P. putida* (overnight culture, OD_600_ ~1.2) were grown in the bottom of the microtiter plate and then exposed to biofilms consisting of *E. coli* MWCBs (pVIBdeltaI and pSB1075) that had formed on the PEG, and the bioluminescence was monitored in a time-dependent manner (Figure 9). With both MWCBs, bioluminescence remained constant at lower concentrations of *P. putida* and was only reduced when higher concentrations than diluted 1/1000 were used at 1 h and 4 h. In contrast, biofilm formation as measured by gramCV staining was reduced by *P. putida* with both MWCBs at much lower concentrations, starting already with 1/10^7^ diluted samples in a concentration-dependent manner, suggesting uncoupling of biofilm formation from quorum sensing.

## 4. Discussion

Microbial biofilm communities can develop during infection in wounds and implantable devices, where they often become more virulent and develop tolerance and resistance. Thus, to identify potential molecular targets for therapy and to understand disease pathogenicity, it is important to study how bacteria exchange information when grown as a community in biofilms. Here, we have developed an assay system that can be used to measure bacterial communication within biofilms. The assay has several advantages when compared to existing biofilm assays that are based on endpoint staining of the biofilm with gram-crystal violet, elution of the dye, and measuring the absorbance [27]. In our assay, bacteria in the biofilm remain alive and can be monitored over time, therefore giving a time-dependent picture of bacterial communication during development, maintenance, and disintegration of the biofilm. Further, the assay takes only minutes for measurement of bioluminescence with a microtiter plate reader, facilitating usage in a high-throughput format that has the potential of being fully automatable. The assay can be arranged in multiple formats; the simplest one is to assess biofilm formation by measuring bioluminescence emitted by a constitutively expressed reporter protein expressed by the bacteria. More elaborate formats assess bacterial communication molecules and the response to stressors by integrating MWCBs designed to detect QSMs, QSIs or stressors into the biofilms. The 96-well microtiter plate format allows for assessing multiple conditions (concentrations, type of treatments and time, as well as number of bacteria) in the same plate for comparison in multiple replicates relevant for assays with high biological variability such as biofilm formation. No elaborate staining, washing, destaining or fixing procedures are required, unlike traditional biofilm assays. Further, we show that interactions within bacterial communities grown as a mixed culture of separate strains can be studied, or one strain as biofilm and the other planktonic. Interactions of two or more species of bacteria can be monitored by using Sender and Receiver bacteria, and if desired, additional fluorescent labels (e.g., green fluorescence protein (GFP), phytochromes (near-infrared), or acridine orange) can be used [80,81].

We have tested several MWCBs for their response to increasing concentrations of QSMs (AHLs and DPO) when integrated into biofilms. As expected, we observed a concentration and time-dependent increase in bioluminescence production, reflecting quorum sensing in the biofilm. Intriguingly, at high concentrations of QSMs, and at extended time of treatment, we observed a decline in bioluminescence, “*Quorum Silencing*”, possibly reflecting that a response to QSMs is not required anymore once mature biofilms are formed. Our assay measures the sum of events occurring upon exposure to QSMs so that the observed phenomenon “Quorum Silencing” may result from several mechanisms, such as metabolic changes upon shifts to different growth phases, induction of dormancy similar to spore formation, the production of inhibitory metabolites such as QSIs, substrate/oxygen depletion, or potential toxicity of QSMs. However, the rapid decline of communication with increasing QSMs concentration and time of exposure (e.g., for AHLs starting at ~50 nM at 24 h) suggests that this is indeed an actively regulated phase of QS that occurs in biofilms. Further, we found that two QSIs, curcumin and DIM, lead to inhibition of quorum sensing in biofilms at higher concentrations after 1 h and 4 h, but this is not followed by biofilm disintegration. It can be envisioned that the high-throughput format of our assays in microtiter plates will facilitate screening of many natural bioactive compounds, drugs and antibiotics and their nano formulations to identify and optimize their effects on quorum sensing and biofilm formation [55,56].

Antibiotics can either be bacteriostatic or bactericidal. One of the mechanisms of bactericidal action of antibiotics involves the induction of oxidative damage by reactive oxygen radicals (ROS) such as ^•^OH generated from H_2_O_2_ and iron by Fenton chemistry [63,64,65,82]. H_2_O_2_ is commonly used as an antiseptic and additive in cleaning agents to more efficiently inactivate bacteria. Intracellular H_2_O_2_ increases in the presence of certain antibiotics [65], and, as a consequence, bacteria have developed systems to defend themselves against oxidants such as H_2_O_2_ and have become resistant [66,67,68]. In our studies, H_2_O_2_ above 0.002% efficiently inhibited the activity of the MWCBs and led to biofilm disintegration. Intriguingly, at lower concentrations of H_2_O_2_, we observed induction of the MWCBs, possibly reflecting a hormetic response and activation of genes important for resistance to stressors such as oxidants and antibiotics [69,83]. In contrast to H_2_O_2_, the antibiotics inhibited the MWCBs activity at concentrations above their minimal inhibitory concentration (MIC), but this did not necessarily lead to the disintegration of the biofilm. Interestingly, like low concentrations of H_2_O_2_, at antibiotic concentrations just below their MIC, we observed induction of communication in the biofilm, possibly as a result of H_2_O_2_ generated during their bactericidal action. This induction of communication could be visualized when the MWCBs were grown in bacterial lawns, with induction of bioluminescence at the border between the “zone of death” and the bacteria that remain alive, a zone where the bacteria may defend themselves against oxidative stress [84]. Further, the observed reduction in communication in response to antibiotics by iron chelators suggests the involvement of H_2_O_2_ and ROS generated by the Fenton reaction. Further measurements using oxygen-sensitive chemical probes that become fluorescent upon exposure to ROS, or fluorescent expression systems that report oxidative damage in living cells, may be required to confirm increased production of ROS in the observed defense zone [85]. So far, we have mainly investigated *E. coli* strain JM109 grown under aerobic conditions, but biofilms can also be formed in our assay system using anaerobic conditions with facultative anaerobic *E. coli* and several types of anaerobic bacteria. However, since the luciferase reporters require oxygen for generation of light, novel sensor plasmids will be required for anaerobic bacteria using reporters that do not need oxygen such as near-infrared fluorescent proteins [81]. Likewise, the assay can be adapted to pathogenic bacteria such as *Helicobacter pylori*, *Pseudomonas aeruginosa* or *Streptococcus mutans* (dental plaques) in which QS can play a role in inducing biofilms and bacterial resistance against antibiotics [56,86,87]. In pathogenic bacteria, the role of QS for biofilm formation is best studied with *P. aeruginosa*, and the possibility of reversing biofilm formation through QS inhibitors and antibiotics has been addressed [69,88,89,90]. These studies indicate that at least in *P. aeruginosa*, subclinical doses of certain antibiotics, such as ciprofloxacin, affect QS-dependent gene expression [86]. At the mechanistic level, these antibiotics did not modulate AHL-synthetase expression, and in silico docking suggested that their effect is independent of binding to LasR but may involve changes in membrane permeability affecting the flux of QSMs [86]. Further, inhibition of communication in biofilms by high concentrations H_2_O_2_ was observed, likely as result of disrupting LasR binding to its target DNA after formation of a disulfide bond between Cys201 and Cys203, and it remains to be shown whether H_2_O_2_ and ROS generated by the Fenton reaction are involved.

Recent research has highlighted the need to understand the physiological role and regulatory mechanisms by which bacteria communicate in biofilms [91]. To live in communities, microbes exchange information with each other that can be investigated using our developed assay system. Communication, quorum sensing, and gene expression are different in bacteria grown in biofilms, in lawns, and when compared to the homogeneous liquid culture with planktonic bacteria, which is a typical method of bacterial culture investigation [31,92,93]. Biofilms are structured and often consist of several different bacterial species that communicate with each other by exchanging QSMs and QSIs, enzymes that metabolize and degrade QSMs or molecules that interfere with signaling by QSMs [94,95,96]. The formation of biofilms can be seen as a community response to escape adverse events, much like spores are formed from single cells for survival, and both processes are modulated by QSMs [97,98,99,100]. In several infectious and chronic diseases, the bacterial community composition is shifted (dysbiosis), but it is still unclear how and to what degree QSMs and QSIs contribute and whether normalization of communication by a change in nutrition/probiotics or by drugs is effective as therapy [101]. An up to 100–1000 times higher concentration of antibiotics and disinfectants is tolerated in biofilms when compared to planktonic cells [4]. Biofilms are thought to be the place where bacteria adapt, persisters tolerant to antibiotics and stressors emerge and survive, and the mechanisms involved can possibly be identified by detecting mutations in resistance genes and gene expression patterns that are altered [5,8,44,69,84].

In fact, whole transcriptome studies with *E. coli* have reported changes in signaling and the expression of genes and small RNAs (sRNA) related to the stress response that can be measured using our assay in future studies [22,102,103,104]. Biofilm formation and recombinant protein expression are affected by the presence of antibiotics and the composition of the growth media [105]. Since the bacteria in our assay remain alive in the biofilm on the PEG, we will isolate total RNA for assessment with transcriptomics/RNAseq to correlate the relationship between communication response, environmental stressors, and antibiotics to changes in genome-wide gene expression. We anticipate that by identifying the optimal position and timepoint at which communication occurs in biofilms in response to stressors, the relevant genes can be identified and assigned a physiological role of the observed communication changes in developing tolerance and resistance (e.g., “*Quorum Silencing*”). These studies will provide cues on how these bacteria in biofilms respond at the transcriptomics level to QSMs, QSIs, and stressors (e.g., oxidants and antibiotics), and how biofilms can be prevented (e.g., in the body and on medical tools or implantable devices), and how persisters tolerant or resistant to antibiotics emerge. In a preliminary transcriptomics analysis with RNA isolated from the defense zone, we have identified genes known to be regulated by stress and that provide stress resistance, showing the feasibility of this approach. Further analysis will be required to demonstrate the physiological role of these genes in providing stress resistance and in generating persisters.

## 5. Conclusions

Using *E. coli* as a host for MWCBs for QSMs, we have developed a high-throughput assay system for measuring communication in bacterial biofilms. The assay is highly adaptable and can be modified in multiple ways with its application to biofilms formed by other bacterial species, different MWCBs, and varying experimental conditions. For the developed pSmut reporter, σ^54^ (RpoN) appeared to be essential, a factor known for its regulatory role in biofilm formation in response to various stress conditions, but for other MWCBs, other sigma factors such as σ^S^ (RpoS) may contribute and modulate the response [61,62,106,107]. In future work, possible influences of bacterial growth phases on the assay system (e.g., early-, mid-, and late-logarithmic phases, as well as stationary/dormant phases) should be investigated, which may affect metabolism and composition of the growth media (nutrients, oxygen, and toxic metabolites) [108]. We envision that a better understanding of the communication mechanisms generated by bacteria in biofilms will provide valuable insights into their interactions and community behavior in natural environments. This study may inform the development of strategies to modulate bacterial communication in response to antibiotics and other stressors.

## Data Availability

The original contributions presented in this study are included in the article/Supplementary Materials. Further inquiries can be directed to the corresponding authors.

## Supplementary Materials

The following supporting information can be downloaded at: https://www.mdpi.com/article/10.3390/antiox15030361/s1, Detailed Methods Section (Construction of novel MWCBs plasmids); Figure S1. Construction of plasmids pSmut and pSmutVIBI; Figure S2. Plasmid pSmut is dependent on σ^54^ (NtrA); Figure S3. Construction and testing of Receiver plasmid pVIBdeltaI; Figure S4. Testing Sender and Receiver plasmids in liquid culture and lawns; Figure S5. Measuring the response of the MWCB to DPO. References [109,110,111,112] are cited in Supplementary Materials.

## Abbreviations

The following abbreviations are used in this manuscript:

AFM	atomic force microscopy
AHLs	N-acetyl-homoserine lactones
BBC	biofilm bactericidal concentration
BPC	biofilm prevention concentration
CLSM	confocal laser scanning microscopy
CV	crystal violet
DIM	3,3′-diindolylmethane
DPO	3,5- dimethylpyrazin-2-ol
eDNA	extracellular DNA
EPS	extracellular polymeric substances
GFP	green fluorescent protein
IBD	inflammatory bowel disease
IPTG	isopropyl-beta-D-thiogalactopyranoside
lcAHLs	long-chain AHLs
LM	light microscopy
LOD	limit of detection
MBC	minimum bactericidal concentration
MBEC	minimum biofilm eradication concentration
MBIC	minimum biofilm inhibitory concentration
MIC	minimal inhibitory concentration
MWCBs	microbial whole cell biosensors systems
QS	quorum sensing
QSIs	Quorum-sensing inhibitors
QSMs	Quorum-sensing molecules
RLUs	relative light units
ROS	reactive oxygen species
scAHLs	short-chain AHLs
sRNA	small RNA
SEM	scanning electron microscopy
