# Supplementary material for: Measuring Communication in Microbial Biofilms in Response to Antibiotics, Phytochemicals and Stressors
Source: Antioxidants (Basel). 2026 Mar 12;15(3):361. doi: 10.3390/antiox15030361 (PMC13024455; doi:10.3390/antiox15030361)
Supplement: Supplementary file 1 [file antioxidants-15-00361-s001.zip › antioxidants-4083258-supplementary.pdf]

Supplementary information to:

Measuring communication in microbial biofilms in response to antibiotics, phytochemicals and stressors

Jean-Marc Zingg[1,2]*, Pratibha Joshi[1], Michael Moraskie[1], Mengrui Li[1], Sherwin Reyes[1,2], Md Harun Or Roshid[1, 2], Sapna Deo[1, 2, 3], Sylvia Daunert[1, 2, 3]*

[1]Department of Biochemistry and Molecular Biology, Miller School of Medicine, University of Miami, Miami, FL 33136-6129, USA

[2]Dr. John T. Macdonald Foundation Biomedical Nanotechnology Institute, University of Miami, Miami, Florida 33146-2101, USA

[3]University of Miami Clinical and Translational Science Institute, University of Miami, Miami, Florida 33136-6129, USA

****Corresponding authors***

Detailed Methods Section

Construction of novel MWCBs plasmids

Plasmid construction of pPsmut for measuring the σ^{54}-dependent stress response. We have engineered and characterized a novel MWCBs, pPsmut, for the detection of the σ^{54}-dependent stress response as result of exposure to oxidants, antibiotics, xylenes and related organic compounds. This MWCB takes advantage of a mutant transcriptional activator of *XylR*, *XylR28*, and a truncated version of the corresponding operator/promoter region taken from the *Xyl* operon from *Pseudomonas putida* (**Figure S1**) [1-4]. The mutated XylR28 protein is under the control of the Pr1/2 promoters and the LuxCDABE cassette from *Photorhabdus luminescence* is under the control of the XylR inducible Ps1 promoter. PCR was performed with plasmid pBBXylR and primer LMdelfw2 (5′-CCA<u>TACGTA</u>ACGG***TTCTCTT***GCCACGTTGCGCCATCC-3′) (containing a SnaBI restriction site (underlined) and Shine Dalgarno translation element (bold italics)) from the Ps2 promoter, the inducible σ^{54}-controlled Ps1 promoter, but the constitutive σ^{70}-controlled Ps2 promoter deleted) and primer LDdelrev2 (5′-CTA<u>CTCGAG</u>**CTA**TCGGCCCATTGCTTTCACAG-3′) containing a XhoI restriction site (underlined) and the stop codon (**bold**) of the XylB28 gene. PCR conditions were 30s 98°C, 10s 98°C, 30s 62°C, 4 min 72°C, 40 cycles. Extension 4 min 72°C using the Q5 High-fidelity DNA polymerase (NEB). This PCR fragment was separated on an agarose gel, isolated, cut with XhoI and SnaBI and ligated with T4 ligase (NEB) into pGEN-LuxCDABE (Addgene) cut with XhoI and SnaBI and transformed into NEB5α. The construct was confirmed by Sanger sequencing. The construct (pPsmut) contains a truncated version of the Ps promoter containing only the σ^{54} controlled Ps1 promoter but not the σ^{70} controlled Ps2 promoter that is regulated by XylR and controls the expression of the luxCDABE reporter cassette.

Plasmid construction of pPsmutdeltaE. During sequencing of the XylR gene from plasmid XylR28, it was noted that it contained an additional EcoRI site at the 5′-end generated during construction that extended the amino acid sequence by two amino acids (GluPhe) [5]. Analysis of the protein structure of XylR28 using Alphafold (version 2.0) revealed that these two additional amino acids may not change the overall structure of XylR28, but it appeared possible that they may affect the A domain that act as an intramolecular repressor and shifts its orientation during activation by ligand binding [6]. Therefore, these two amino acids were deleted using PCR and primers Xyldelfw: 5′-p-CATTTGTTTTCCTCTTGTTTTTATCGGG-3′ and Xyldelrv: 5′-p-TCGCTTACATACAAACCCAAGATG-3′. PCR conditions were 10s 98°C, 10s 98°C, 30s 55°C, 7 min 72°C, 40 cycles and final extension 4 min 72°C using the Q5 High-fidelity DNA polymerase (NEB). The PCR fragments were separated on an 1% agarose gel, isolated and religated using

the KLD kit (NEB). Like pPsmut, pPsmutdeltaE allows for detection of the σ^{54}-dependent stress response, but generally gives a higher output in bioluminescence.

Plasmid construction of pVIBdeltaI. The luciferase cassette of *Aliivibrio fischeri* (plasmid pJE202, [7]) was amplified with forward primer LuxCfw: 5′-p-ATGAATAAATGTATTCCAATGATAATTAATGG-3′ and reverse primer LuxCrv: 5′-p-ACCAACCTCCCTTGCGTTTATTC-3′. The PCR conditions were 10s 98°C, 10s 98°C, 10s 55°C, 6 min 72°C, 40 cycles and final extension 1 min 72°C using the Q5 High-fidelity DNA polymerase (NEB). The resulting PCR fragment was separated on an agarose gel, isolated, and ligated with T4 ligase (NEB) and transformed into NEB5α. The construct was confirmed by Sanger sequencing. The construct contains the *Aliivibrio fischeri* luciferase operon (LuxCDABEG) without LuxI and is inducible by AHLs via LuxR (**Figure S2A**).

Plasmid construction of pSmutVIBI. The LuxI gene of *Aliivibrio fischeri* (plasmid pJE202, [7]) was amplified with forward primer LuxIfw1: 5′-CTTCTACGTATGACTATAATGATAAAAAAATCG-3′ (SnaI underlined) and reverse primer LuxIrv1: 5′-CTTCGCGGCCGCCAACATTAATTTAAGACTGCTTTTTTAAAC-3′ (NotI underlined). The PCR conditions were 10s 98°C, 10s 98°C, 10s 55°C, 1 min 72°C, 40 cycles and final extension 1 min 72°C using the Q5 High-fidelity DNA polymerase (NEB). The resulting PCR fragment was separated on an agarose gel, isolated and cut with SnaI/NotI, and ligated with T4 ligase (NEB) and transformed into NEB5α into pSmut (SnaI/NotI) and pSmutdeltaEcoRI (SnaI/NotI). The construct was confirmed by Sanger sequencing. The construct contains the *Aliivibrio fischeri* LuxI gene expressed by the Ps promoter of plasmid pSmut (**Figure S3**).

Plasmid construction of pETVIBI. PCR with plasmid pJE202 was performed with primers VIBIpETfw: 5′-ATACAT**ATG**ACTATAATGATAAAAAAATCGG-3′ (NdeI underlined, ATG bold) and LuxIrv1: 5′-CTTCGCGGCCGCCAACATTAATTTAAGACTGCTTTTTTAAAC-3′ (NotI underlined). PCR conditions were 30s 98°C, 10s 98°C, 30s 55°C, 1 min 72°C, 40 cycles and final extension 4 min 72°C using the Q5 High-fidelity DNA polymerase (NEB). The resulting PCR fragment (582 bp) was cut with NdeI and NotI, separated by an agarose gel, isolated and then ligated with T4 ligase (NEB) into pET29b(+) that was also cut with NdeI and Not and dephosphorylated by quick CIP (NEB) and transformed into NEB5α. The construct was confirmed by Sanger sequencing. The construct contains the *Aliivibrio fischeri* LuxI gene controlled by the lac-operator repressed T7 promoter and can be induced by isopropyl-beta-D-thiogalactopyranoside (IPTG) (**Figure S3**).

Plasmid construction of pSigma54. The *ntrA* gene (σ^{54}) (P0A171) was amplified from *Pseudomonas putida* KT2440 (ATCC® 47054™) genomic DNA using NtrAfw: 5′-TTAGAATTCACTGGTCAAAGAAGTTTACC-3′ (EcoR underlined) and NtrArv: 5′-TTAAGTACTGTGCATAAAGAGGCAGGTC-3′ (ScaI underlined). PCR conditions were 30s 98°C, 10s 98°C, 30s 62°C, 4 min 72°C, 40 cycles, a final extension 4 min 72°C using the Q5 High-fidelity DNA polymerase (NEB). This PCR fragment was separated on an agarose gel, isolated, cut with EcoRI and ScaI and ligated with T4 ligase (NEB) into pBR322 (NEB) cut with EcoRI and ScaI and transformed into NEB5α. We chose pBR322 as the plasmid with a p15A origin of replication to be compatible when transformed into the same host with the pGEN-LuxCDABE derived plasmids that are based on ColE1 origin of replication. The construct was confirmed by Sanger sequencing (and showed that the EcoRI site got deleted during cloning of a ScaI/ScaI fragment). The construct contains the *Pseudonomas putida ntrA* gene (σ^{54}) expressed under control of its own promoter.

Construction of σ^{54}-responsive plasmids pSmut and pPsmutVIBI

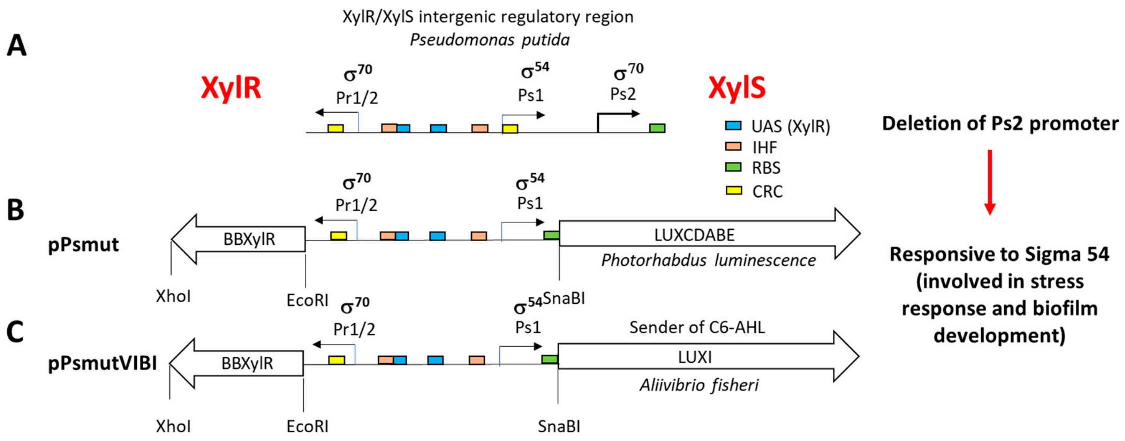

Figure S1. Construction of plasmids pSmut and pSmutVIBI. (**A**) Scheme of the intergenic region of the *Xyl* operon from *Pseudomonas putida*. (**B**) Deletion of the σ^{70}-responsive Ps2 promoter leads to plasmid pSmut that is dependent on σ^{54} to induce the bioluminescence from LuxCDABE from *Photorhabdus luminescence.* (**C**) Replacement of the LUXCDABE cassette of pSmut with LuxI from *Aliivibrio fisheri* produces C6-AHL in response to activation of σ^{54} (e.g. by stressors) (see methods for construction details).

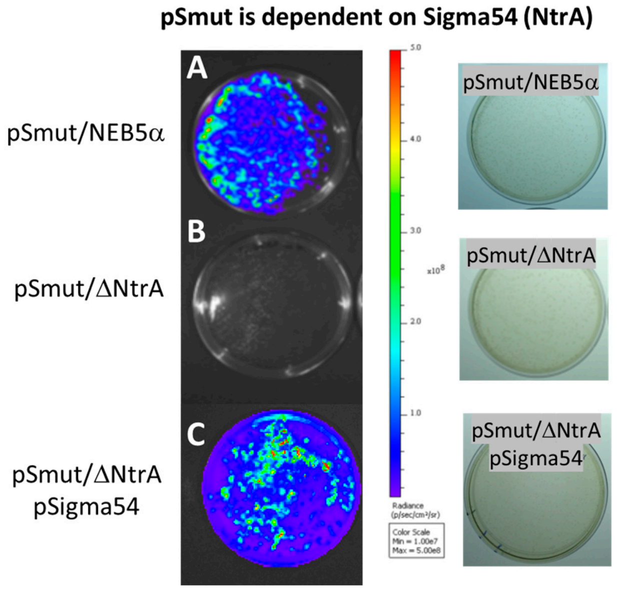

Figure S2. Plasmid pSmut is dependent on σ^{54} (NtrA). (**A**) Colonies of *E. coli* NEB®5α with plasmid pSmut emit bioluminescence on agar plates. (**B**) Plasmid pSmut transformed into σ^{54}-deficient *E. coli* (ΔrpoN730::kan(Δs^{54}) does not emit bioluminescence, unless (**C**), co-transformed with pSigma54 that overexpresses σ^{54} (NtrA). Left pictures are acquired by IVIS; Right pictures are acquired by camera.

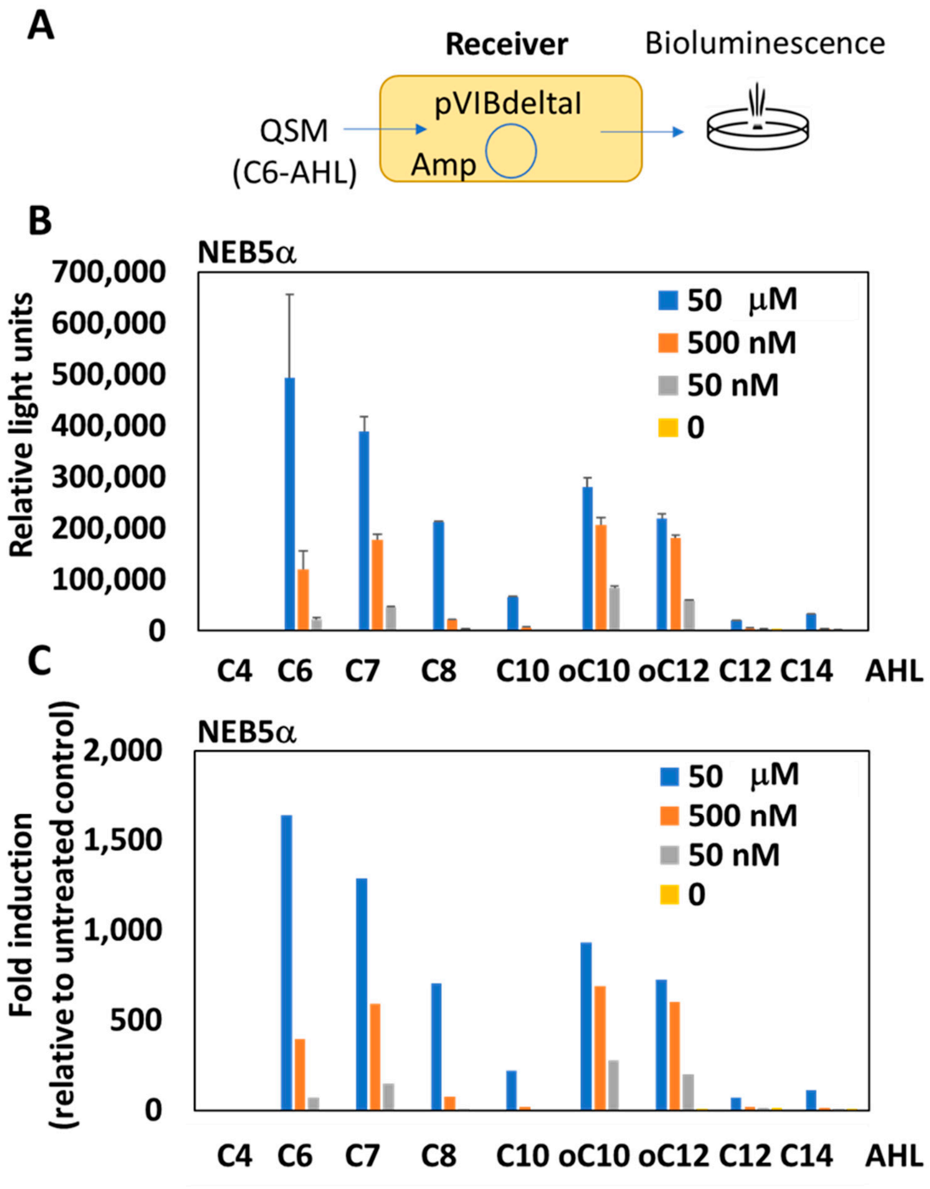

Figure S3. Construction and testing of Receiver plasmid pVIBdeltaI. (**A**) The Receiver bacteria with plasmid pVIBdeltaI become bioluminescent in response to C6-AHL (**B and C**) Induction of pVIBdeltaI with decreasing concentrations of different types of AHLs (C4, C6, C8, C10 oxoC10, oxoC12, C12, C14-AHL). (**B**) relative light units; (**C**) fold induction relative to untreated control. Note that in the absence of AHLs, no background bioluminescence is measured (in orange, **A**, **B**).

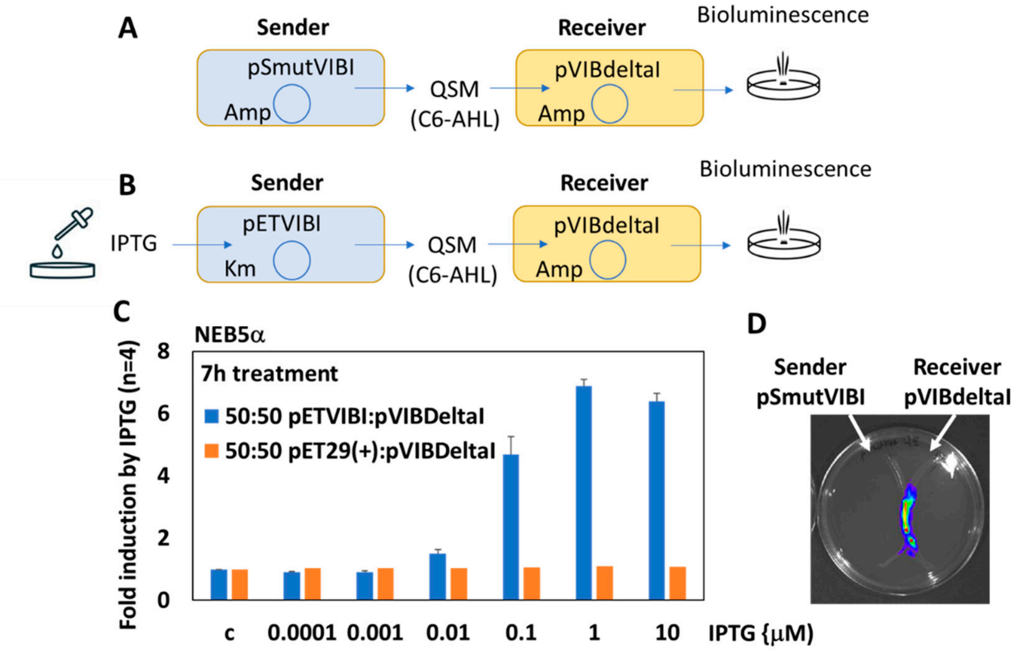

Figure S4. Testing Sender and Receiver plasmids in liquid culture and lawns. (**A**) The Sender bacteria containing plasmid pSmutVIBI synthesize C6-AHL upon activation of σ^{54} (e.g., by stressors), leading to activation of the Receiver bacteria containing plasmid pVIBdeltaI and emission of bioluminescence. (**B**) The Sender bacteria containing plasmid pETVIBI produce C6-AHL upon induction by IPTG, and activate the Receiver bacteria containing plasmid pVIBdeltaI and production of bioluminescence. (**C**) Induction of bioluminescence by Receiver bacteria containing pVIBDeltaI and the Sender bacteria containing plasmid pETVIBI (50:50 mix) with increasing concentrations of IPTG in liquid culture, but not with empty vector pET29(+). (**D**) Induction of the Receiver bacteria containing plasmid pVIBdeltaI when in contact with the Sender bacteria containing pSmutVIBI on agar plates.

Measurement of the response to 3,5-dimethylpyrazin-2-ol (DPO) in bacterial biofilms

3,5- Dimethylpyrazin-2-ol (DPO) is a novel QSM employed by the pathogenic bacterium *Vibrio cholerae* to inhibit biofilm formation and to facilitate spreading [8]. Interestingly, DPO has also been detected in *E. coli* where it is induced by the translational stressor erythromycin, but its regulatory functions are not yet clear [9-13]. DPO is a structural isomer of the QSM autoinducer 3 (AI3) that is also produced in *E. coli* but does not activate the DPO MWCBs and therefore we use AI3 as control [9]. In a recent study we have constructed and characterized a MWCBs to enable DPO detection [9]. The constructed plasmid pSD8693-BBa was transformed into *tdh*-knockout *E. coli* strains (SP942, JW3591-4) that do not produce DPO.

Response to treatment with DPO and AI3 when grown in biofilms

We formed biofilm by growing the MWCB for DPO on the PEG of the microtiter plate (plasmid pSD8693-BBa was transformed into *tdh*-knockout *E. coli* strains (SP942, JW3591-4)). The treatment of pSD8693-BBa with DPO slightly induced bioluminescence in the biofilm (**Fig S5A**). We observed a decrease of bioluminescence with DPO at high concentrations (>10 nM), "*Quorum Silencing*", but much less with the structural isomer, autoinducer 3, AI3 (which is not recognized by the MWCBs and is used as control) (**Figure S5B**). With increasing concentrations of DPO and less with AI3, biofilm formation is increased, suggesting that these QSMs may have a role in *E. coli* biofilm formation what remains to be further investigated.

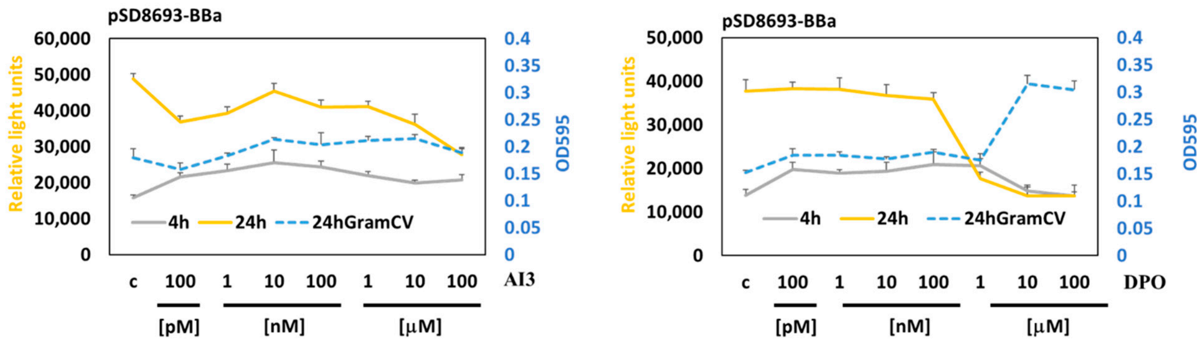

Figure S5. Measuring the response of the MWCB to DPO. The MWCBs for DPO (pSD8693) was grown as biofilm using the MBEC assay microtiter plates and treated with increasing concentrations of AI-3 (**A**) or DPO (**B**) and the bioluminescence measured at 1h, 4h and 24h. GramCV staining (OD_{595}) was used to measure the biofilm integrity after treatment for 24h (blue stippled lines).

References

1. Moreno, R.; Fonseca, P.; Rojo, F. The Crc global regulator inhibits the Pseudomonas putida pWW0 toluene/xylene assimilation pathway by repressing the translation of regulatory and structural genes. *J Biol Chem* **2010**, *285*, 24412-24419, doi:10.1074/jbc.M110.126615.
2. de Las Heras, A.; de Lorenzo, V. Cooperative amino acid changes shift the response of the sigma(5)(4)-dependent regulator XylR from natural m-xylene towards xenobiotic 2,4-dinitrotoluene. *Mol Microbiol* **2011**, *79*, 1248-1259, doi:10.1111/j.1365-2958.2010.07518.x.

3. Zingg, J.M.; Daunert, S. From Quorum Sensing to Positional Sensing. *The Faseb Journal* **2020**, *34*, 1, doi:https://doi.org/10.1096/fasebj.2020.34.s1.02508.
4. Zingg, J.-M.; Joshi, P.; Zhu, Y.; Deo, S.; Daunert, S. Abstract 1815: Development of Whole Cell Biosensor Systems for Positional Biosensing in Bacterial Biofilms. *Journal of Biological Chemistry* **2023**, *299*, 103118 https://linkinghub.elsevier.com/retrieve/pii/S0021925823003903.
5. de las Heras, A.; Carreno, C.A.; de Lorenzo, V. Stable implantation of orthogonal sensor circuits in Gram-negative bacteria for environmental release. *Environ Microbiol* **2008**, *10*, 3305-3316, doi:10.1111/j.1462-2920.2008.01722.x.
6. Perez-Martin, J.; De Lorenzo, V. The amino-terminal domain of the prokaryotic enhancer-binding protein XylR is a specific intramolecular repressor. *Proc Natl Acad Sci U S A* **1995**, *92*, 9392-9396.
7. Engebrecht, J.; Silverman, M. Identification of genes and gene products necessary for bacterial bioluminescence. *Proc Natl Acad Sci U S A* **1984**, *81*, 4154-4158, doi:10.1073/pnas.81.13.4154.
8. Silpe, J.E.; Duddy, O.P.; Papenfort, P. Microbial Communication via Pyrazine Signaling: A New Class of Signaling Molecules Identified in Vibrio cholerae. *Israel Journal of Chemistry* **2023**, *63*, 1-11.
9. Moraskie, M.; Roshid, M.H.O.; O'Connor, G.; Artola Zavala, T.; Dikici, E.; Zingg, J.M.; Deo, S.; Daunert, S. Engineered biosensors for the quorum sensing molecule 3,5-dimethyl-pyrazine-2-ol (DPO) reveal its presence in humans, animals, and bacterial species beyond Vibrio cholerae. *Biosens Bioelectron* **2023**, *237*, 115494, doi:10.1016/j.bios.2023.115494.
10. Gatsios, A.; Kim, C.S.; York, A.G.; Flavell, R.A.; Crawford, J.M. Cellular Stress-Induced Metabolites in Escherichia coli. *J Nat Prod* **2022**, *85*, 2626-2640, doi:10.1021/acs.jnatprod.2c00706.
11. Kim, C.S.; Gatsios, A.; Cuesta, S.; Lam, Y.C.; Wei, Z.; Chen, H.; Russell, R.M.; Shine, E.E.; Wang, R.; Wyche, T.P.; et al. Characterization of Autoinducer-3 Structure and Biosynthesis in E. coli. *ACS Cent Sci* **2020**, *6*, 197-206, doi:10.1021/acscentsci.9b01076.
12. Papenfort, K.; Silpe, J.E.; Schramma, K.R.; Cong, J.P.; Seyedsayamdost, M.R.; Bassler, B.L. A Vibrio cholerae autoinducer-receptor pair that controls biofilm formation. *Nat Chem Biol* **2017**, *13*, 551-557, doi:10.1038/nchembio.2336.
13. Mashruwala, A.A.; Bassler, B.L. The Vibrio cholerae Quorum-Sensing Protein VqmA Integrates Cell Density, Environmental, and Host-Derived Cues into the Control of Virulence. *mBio* **2020**, *11*, doi:10.1128/mBio.01572-20.
